# Immunogenomic Profiling Demonstrate AC003092.1 as an Immune-Related eRNA in Glioblastoma Multiforme

**DOI:** 10.3389/fgene.2021.633812

**Published:** 2021-03-18

**Authors:** Xiao-Yu Guo, Sheng Zhong, Zhen-Ning Wang, Tian Xie, Hao Duan, Jia-Yu Zhang, Guan-Hua Zhang, Lun Liang, Run Cui, Hong-Rong Hu, Jie Lu, Yi Wu, Jia-Jun Dong, Zhen-Qiang He, Yong-Gao Mou

**Affiliations:** ^1^Department of Neurosurgery/Neuro-oncology, State Key Laboratory of Oncology in South China, Collaborative Innovation Center for Cancer Medicine, Sun Yat-sen University Cancer, Guangzhou, China; ^2^The First Clinical Medical College of Yunnan University of Traditional Chinese Medicine, Kunming, China; ^3^Department of Cerebrovascular Surgery, The Third Affiliated Hospital, Sun Yat-sen University, Guangzhou, China; ^4^Department of Neurosurgy, Jiangmen Central Hospital, Jiangmen, China

**Keywords:** lncRNA, eRNA, glioblastoma multiforme, AC003092.1, TFPI2, immunogenomic

## Abstract

Enhancer RNAs, a type of long non-coding RNAs (lncRNAs), play a critical role in the occurrence and development of glioma. RNA-seq data from 161 glioblastoma multiforme (GBM) samples were acquired from The Cancer Genome Atlas database. Then, 70 eRNAs were identified as prognosis-related genes, which had significant relations with overall survival (log-rank test, *p* < 0.05). AC003092.1 was demonstrated as an immune-related eRNA by functional enrichment analysis. We divided samples into two groups based on AC003092.1 expression: AC003092.1 High (AC003092.1_H) and AC003092.1 Low (AC003092.1_L) and systematically analyzed the influence of AC003092.1 on the immune microenvironment by single-sample gene-set enrichment analysis and CIBERSORTx. We quantified AC003092.1 and TFPI2 levels in 11 high-grade gliomas, 5 low-grade gliomas, and 7 GBM cell lines. Our study indicates that AC003092.1 is related to glioma-immunosuppressive microenvironment, and these results offer innovative sights into GBM immune therapy.

## Introduction

In the human central nervous system, gliomas are the most common primary tumor. Among gliomas, glioblastoma multiforme (GBM) is the most malignant and has the worst prognosis. Despite that surgery, radiotherapy, and chemotherapy were applied, the treatment effect of patients with glioblastoma is still worse (Cuddapah et al., 2014). So far, the median survival time for patients with glioblastoma is only 14 months (Weller et al., 2015).

Long non-coding RNAs (lncRNAs) are a type of non-coding RNAs longer than 200 nucleotides (Wapinski and Chang, 2011; Spizzo et al., 2012). LncRNAs have multiple functions such as chromatin topology, scaffolding and modulating the activity of proteins and RNA, regulators of neighboring transcription, and encoding functional micropeptides (Ransohoff et al., 2018), and part of lncRNAs has been shown to encode functional peptides or proteins (Matsumoto et al., 2017; Rion and Ruegg, 2017; Lu et al., 2019; Cardon et al., 2020). With the identification of more and more functional lncRNAs, lncRNAs have become a critical factor in the occurrence and development of gliomas (Peng et al., 2018).

Enhancer RNAs (eRNAs), a subclass of lncRNAs, are transcribed from enhancers and regulate biological process by adjusting target gene expression (Long et al., 2016). Thousands of eRNAs have been determined to play an important role in the transcription of human cells (Li et al., 2016). During the development of malignant tumors, eRNA can participate in the expression of oncogenes and the activation of oncogenic pathways. In prostate cancer, KLK3e, an androgen-induced eRNA, scaffolds the androgen receptor (AR)–associated protein complex could modulate chromosomal architecture and enhance AR-dependent KLK3 expression to promote prostate carcinogenesis (Hsieh et al., 2014). And in regulation of the immune response, eRNA (IL1β-eRNA) attenuates messenger RNA transcription and release of the proinflammatory mediators to influence human innate immune response (IIott et al., 2014). As far as we know, the researches of immune-related eRNA in GBM have not been reported.

In our study, we identified prognosis-related eRNAs and their predicted target genes in GBM. According to our analysis, lncRNA *AC003092.1* was an immune-related eRNA with significant correlation to survival in patients with GBM and upregulated target gene, *TFPI2* (tissue factor pathway inhibitor 2), expression. Immunogenomic analysis showed that the expression of *AC003092.1* is related to the immune cell fractions, function, and pathways. We validated the positive correlation between the expression of *AC003092.1* and *TFPI2* in high-grade gliomas and glioblastoma cell lines.

## Materials and Methods

### Gene Expression Datasets and Data Processing

**FIGURE 1:**
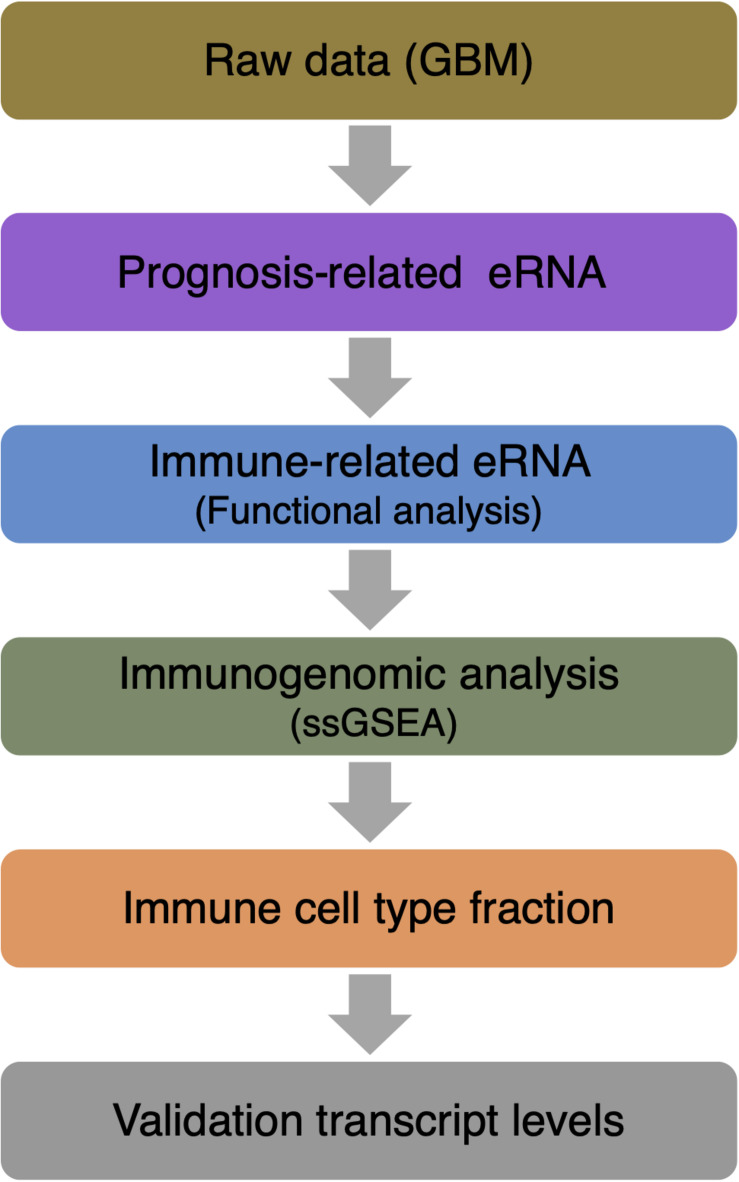
Data analysis workflow.

RNA-seq data of 161 GBM patients were obtained from The Cancer Genome Atlas (TCGA) datasets. All patients had accurate follow-up data. For further analysis, RNA transcriptome profiling was then performed using log2-based transformation of FPKM values by R Studio software (version 1.2.5001). Figure 1 shows the paths of the data analysis.

### Identification of Prognosis-Related eRNA in GBM

PreSTIGE, an algorithm that could predict tissue-specific enhancers based on the H3K4me1 mark and tissue-specific expression of mRNAs, was applied to predict lncRNAs expressed from active tissue-specific enhancers and their target genes (Corradin et al., 2014; Vucicevic et al., 2015). Ensembl transcript ID and gene symbol were annotated by “biomaRt” R package (human GRCh38.p13) (Durinck et al., 2009; Smedley et al., 2009). The log-rank test was used to calculate the differences between eRNA expression (cutoff value = 50%) and survival time in GBM patients using a threshold of *p* < 0.05. Based on the result of log-rank test, Kaplan–Meier curves were plotted to indicate the survival time differences. According to the list of eRNAs and target genes, Spearman correlation test was used to evaluate correlations and Spearman rank correlation coefficient *r* > 0.4, *p* < 0.001 as the threshold.

### Functional Enrichment Analysis

All transcripts with significant correlation (Spearman rank correlation coefficient *r* > 0.4, *p* < 0.001) with prognosis-related eRNAs were identified as eRNA-related genes by Spearman correlation test. To investigate gene ontology and signaling pathway enrichment, eRNA-related genes were uploaded to online tool Metascape^1^ website for gene annotation, functional enrichment, and interactome analysis (Ashburner et al., 2000; Lebrec et al., 2009; Gene Ontology Consortium, 2015; Zhou et al., 2019). The terms were considered as significant and grouped into clusters when *p* < 0.01 and the numbers of enriched genes ≥3 based on their membership similarities. According to results of functional enrichment analysis, we confirmed the immune-related eRNA for further research.

### Immunogenomic Analysis of AC003092.1

One hundred sixty-one GBM patients were divided into two groups, 80 AC003092.1_H patients and 81 AC003092.1_L patients, according to the expression of *AC003092.1* (cutoff value = 50%). In every GBM patient, the single-sample gene-set enrichment analysis (ssGSEA) score was used to measure the enrichment levels of the 29 immune signatures (Supplementary Table 3) (Barbie et al., 2009; Hanzelmann et al., 2013). The infiltration of immune cell (immune score), stromal content (stromal score), and tumor purity were quantified by Estimation of STromal and Immune cells in MAlignant Tumors using Expression data (ESTIMATE) (Yoshihara et al., 2013). The immunogenomic analysis was executed by R Studio software (version 1.2.5001).

### Assessment of Immune Cell–Type Fractions

The CIBERSORTx and the LM22 gene signature were used to estimate the fraction of immune cell in the GBM patients (Newman et al., 2019). CIBERSORTx^2^ is an analytical tool from the Alizadeh Lab and Newman Lab to impute gene expression profiles and provide an estimation of the abundances of member cell types in a mixed cell population, using gene expression data. We uploaded RNA-seq data as the Mixture file and choose LM22 (22 immune cell types) as Signature matrix file. The successful deconvolution of a sample should be satisfied with the criteria of 1,000 permutations and *p* < 0.05. Mann–Whitney *U* test was used to compare the proportions of the immune cell phenotypes between AC003092.1_H and AC003092.1_L.

### Quantification of AC003092.1 and TFPI2 Transcript Levels in Glioma Samples and GBM Cell Lines

To confirm the RNA-sequencing data, we validated *AC003092.1* and *TFPI2* transcript levels in glioma samples and GBM cell lines by reverse transcription quantitative polymerase chain reaction (RT-qPCR). The isolated total RNA was sourced in 11 HGG patients and 5 LGG patients who were diagnosed at our institution in Sun Yat-sen University Cancer Center (SYSUCC) from February 1, 2019, to July 1, 2019 (Supplementary Table 4), and 7 glioblastoma cell lines (U251, U138, T98G, SKMG4, U87, U118, and LN229). U251, U138, T98G, SKMG4, U87, U118, and LN229 were obtained from the State Key Laboratory of Oncology in South China and were maintained in Dulbecco modified eagle medium supplemented with 10% fetal bovine serum. All cells were maintained in a humidified incubator at 37°C and 5% CO_2_. The Ethics Committee of Sun Yat-sen University Cancer Center approved the procedures (no. GZR2018-244). Total RNA was isolated from tissue samples or cultured cells using RNA-Quick Purification Kit (ESscience, Shanghai, China). The cDNA was synthesized from 1,000 ng total RNA by Fast All-in-One RT Kit (ESscience, Shanghai, China). RT-qPCR was performed by Bio-Rad CFX96 Real-Time PCR System (Bio-Rad Laboratories, Inc., Hercules, CA, United States) and LightCycler 480 II Real-Time PCR System (Roche, Basel, Switzerland) with PowerUp SYBR Green Master Mix (Thermo Fisher Scientific, Waltham, MA, United States). Custom primers for AC003092.1 (forward: TTAGCAGCAAACCCAGAAC, reve rse: TGCTGAGGATACATGACGAA) and TFPI2 (forward: GCCTGAGAACTTTGAATGATGCTG, reverse: GGCCCTGT GTTTCTTATGTATCCTG) were obtained from Tianyi Huiyuan Bioscience and Technology Inc., Wuhan, China. Each sample was measured in triplicate, and the results were normalized to the housekeeping gene U6 (forward: CTCGCTTCGGCAGCACA, reverse: AACGCTTCACGAATTTGCGT) and GAPDH (for ward: GGAGCGAGATCCCTCCAAAAT, reverse: GGCTGTT GTCATACTTCTCATGG).

## Results

### Analysis of Prognosis-Related eRNA in GBM

According to previous study, there are 2,695 lncRNA transcripts, and 2,303 predicted target genes have been identified by the PreSTIGE algorithm (Vucicevic et al., 2015). The eRNA-target genes pairs were confirmed by this eRNA transcripts database. For further analysis, we conversed transcript ID to gene ID by “biomaRt” R package (Durinck et al., 2009). After conversion, the 2,695 eRNA transcripts were mapped to 1,288 genes. Then log-rank test was used to analyze the relationship of eRNAs and over survival in RNA-sequence data from 161 GBM samples of TCGA database. Next, we identified that 70 eRNAs were significantly associated with overall survival (Supplementary Table 1, log-rank test, *p* < 0.05).

Finally, the correlation between eRNAs and their predicted target genes was evaluated, and only 28 eRNAs were included in the next study (Spearman rank correlation coefficient *r* > 0.4, *p* < 0.001; Table 1).

**TABLE 1:** List of eRNAs-target gene pairs with significant correlation.

Ensembl ID	Symbol	Predicted target	Correlation between	
			eRNA and target gene	
			Correlation coefficient *r*	*p* value
ENSG00000224081	*SLC44A3-AS1*	*SLC44A3*	0.71047562	0
		*CNN3*	0.55370945	0
		*F3*	0.44193984	6.39E-09
ENSG00000230461	*PROX1-AS1*	*PROX1*	0.51493846	0
ENSG00000186056	*MATN1-AS1*	*MATN1*	0.47032724	4.58E-10
ENSG00000230402	*LINC01349*	*VCAM1*	0.4614477	7.24E-10
ENSG00000237980	*LINC02773*	*BATF3*	0.53947507	1.54E-13
ENSG00000232973	*CYP1B1-AS1*	*CYP1B1*	0.60617955	0
ENSG00000249307	*LINC01088*	*PAQR3*	0.43993559	7.60E-09
		*NAA11*	0.72891919	5.89E-28
ENSG00000249896	*LINC02495*	*JAKMIP1*	0.85887401	4.62E-48
		*WFS1*	0.47094143	2.89E-10
ENSG00000232021	*LEF1-AS1*	*LEF1*	0.5998476	0
ENSG00000231185	*SPRY4-AS1*	*SPRY4*	0.52109788	0
ENSG00000248859	*LINC01574*	*SNCB*	0.49949671	1.53E-11
		*UNC5A*	0.45996451	8.34E-10
ENSG00000237742	*AL365259.1*	*HEY2*	0.4490942	3.41E-09
ENSG00000232618	*AL355304.1*	*HIVEP2*	0.49455303	2.60E-11
ENSG00000136213	*CHST12*	*IQCE*	0.54750115	0
ENSG00000232930	*AC083864.2*	*EEPD1*	0.59304633	1.14E-16
ENSG00000236453	*AC003092.1*	*TFPI2*	0.59621591	7.16E-17
ENSG00000253824	*AP003472.2*	*RNF19A*	0.45265526	1.65E-09
ENSG00000236924	*AL162411.1*	*GLDC*	0.58796584	0
ENSG00000254605	*AP003555.1*	*ANO1*	0.542206	1.10E-13
ENSG00000255521	*AL356215.1*	*CD44*	0.6796834	3.67E-23
ENSG00000254810	*AP001189.3*	*LRRC32*	0.73750898	6.67E-29
ENSG00000228630	*HOTAIR*	*HOXC10*	0.83311395	9.65E-43
		*HOXC11*	0.91198659	2.04E-63
		*HOXC13*	0.82528477	2.65E-41
		*HOXC6*	0.67094596	2.08E-22
ENSG00000251151	*HOXC-AS3*	*HOTAIR*	0.80140219	2.54E-37
		*HOXC11*	0.82631259	1.73E-41
		*HOXC13*	0.7446111	1.03E-29
		*HOXC6*	0.74653909	6.16E-30
ENSG00000245694	*CRNDE*	*IRX5*	0.73179779	0
ENSG00000232677	*LINC00665*	*ZFP14*	0.5952841	0
		*ZFP82*	0.47718254	2.23E-10
		*ZNF146*	0.4823729	1.25E-10
		*ZNF260*	0.72020646	0
ENSG00000213742	*ZNF337-AS1*	*NINL*	0.40530826	1.26E-07
ENSG00000230736	*AL021937.1*	*RFPL3*	0.45212332	7.65E-10
ENSG00000228274	*AL021707.2*	*GTPBP1*	0.4013285	1.71E-07

### LncRNA AC003092.1 Is an Immune-Related eRNA in GBM

**FIGURE 2:**
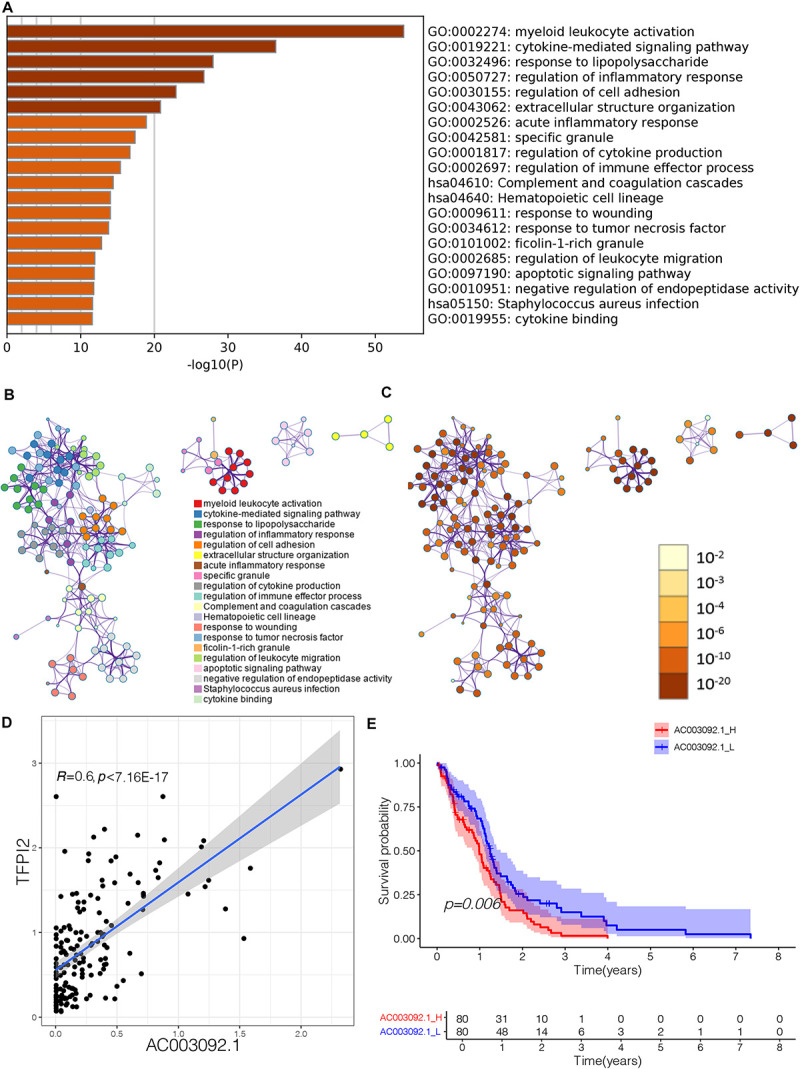
Effect of lncRNA *AC003092.1*, as an immune-related gene, in GBM. **(A)** Heatmap of enriched terms across input gene lists, colored by *p* values. **(B)** Enriched terms colored by cluster ID, where nodes that share the same cluster ID are typically close to each other in *AC003092.1*-related genes. **(C)** Enriched terms colored by *p* value, where terms containing more genes tend to have a more significant *p* value in *AC003092.1*-related genes. **(D)** Scatterplot showing the correlation between *AC003092.1* and *TFPI2* levels. **(E)** Comparison of overall survival between AC003092.1_H and AC003092.1_L by log-rank test.

To further evaluate biological process that eRNAs may regulate, we uploaded the eRNA-related genes to the website Metascape to identify Gene Ontology (GO) terms and Kyoto Encyclopedia of Genes and Genomes (KEGG) pathways. GO terms and KEGG pathways functional enrichment analysis showed that 420 genes that were significantly correlated to lncRNA *AC003092.1* enriched notably in immune-related GO terms (Supplementary Table 2 and Figure 2A). The terms were considered as significant and grouped into clusters when *p* < 0.01 and the numbers of enriched genes were ≥ 3 based on their membership similarities. Each node represents an enriched term and is first colored by its cluster ID (Figure 2B) and then by its *p* value (Figure 2C). In glioblastoma patients, the *AC003092.1* showed a positive correlation with the gene expression of its predicted target gene *TFPI2* (tissue factor pathway inhibitor 2) (Figure 2D, Spearman *r* = 0.6, *p* < 0.001). A previous study had shown that AC003092.1 could upregulate the expression of TFPI2 by inhibiting the function of miR-195 (Xu et al., 2018). According to the expression of *AC003092.1*, the patients were divided into two groups: AC003092.1 High (AC003092.1_H) and AC003092.1 Low (AC003092.1_L) (cutoff value = 50%). Survival analyses showed that the AC003092.1_L had a better prognosis than the AC003092.1_H (Figure 2E, log-rank test, *p* = 0.006).

### Immunogenomic Analysis Between AC003092.1_H and AC003092.1_L

**FIGURE 3:**
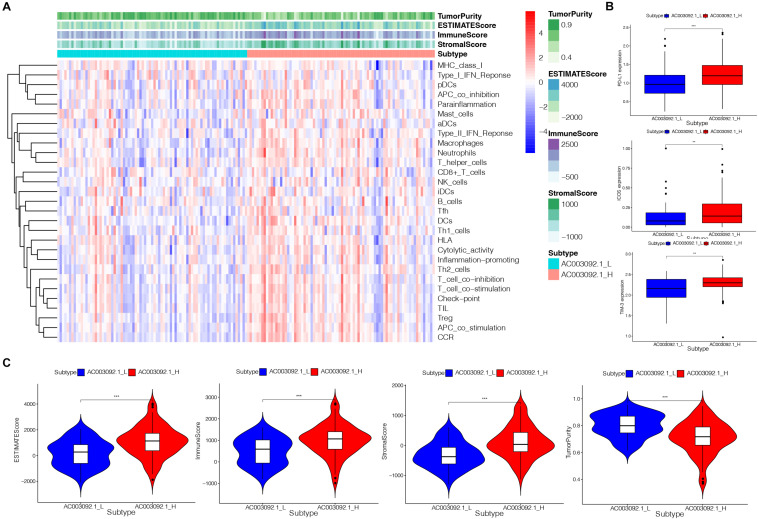
Immunogenomic analysis of AC003092.1_H and AC003092.1_L from TCGA. **(A)** The enrichment levels of the 29 immune signatures by ssGSEA score in each GBM sample. **(B)** Comparison of immune checkpoint gene expression levels between AC003092.1_H and AC003092.1_L (ANOVA test). **(C)** Comparison of the immune score, stromal score, ESTIMATE score, and tumor purity between AC003092.1_H and AC003092.1_L (Kruskal–Wallis test). *p < 0.05, **p < 0.01, ***p < 0.001.

To confirm the immune-related feature between AC003092.1_H and AC003092.1_L, we analyzed 29 sets of immune-associated genes (Supplementary Table 3) representing different immune cell types, functions, and pathways in each GBM patient by ssGSEA analysis (Barbie et al., 2009; Hanzelmann et al., 2013). The results of ssGSEA indicated that immune cell types, functions, and pathways were enriched in AC003092.1_H (Figure 3A). Although the AC003092.1_H had a stronger immune function, the overall survival is better in AC003092.1_L. So, we further confirm the expression of immune checkpoints. We found that PD-L1, ICOS, and TIM-3 expressed highly in AC003092.1_H [analysis of variance (ANOVA) test, *p* < 0.05] (Figure 3B). Therefore, CD8^+^ T cells in AC003092.1_H were exhausted, and AC003092.1_H patients might get more benefit from immune checkpoint inhibitors.

Moreover, the immune scores, stromal scores, ESTIMATE scores, and tumor purity were compared between AC003092.1_H and AC003092.1_L by ESTIMATE. The results showed that the immune scores (Kruskal–Wallis test, *p* < 0.001), ESTIMATE scores (Kruskal–Wallis test, *p* < 0.001), and stromal scores (Kruskal–Wallis test, *p* < 0.001) were higher in the AC003092.1_H group. However, the tumor purity was higher in the AC003092.1_L group (Kruskal–Wallis test, *p* < 0.001) (Figure 3C). From the above, there were more immune and stromal cells in AC003092.1_H, whereas there were more tumor cells in AC003092.1_L.

### The Landscape of Infiltrating Immune Cells in AC003092.1_H and AC003092.1_L

**FIGURE 4:**
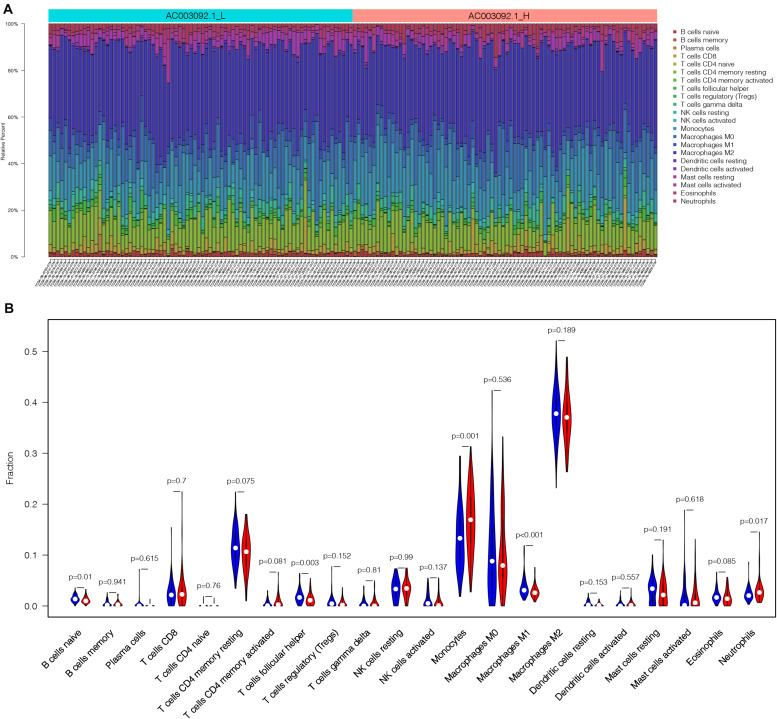
The landscape of infiltrating immune cells in AC003092.1_H and AC003092.1_L. **(A)** The bar plot of immune infiltration between AC003092.1_L and AC003092.1_H. **(B)** Comparison of the immune cell proportion between AC003092.1_H and AC003092.1_L by CIBERSORTx (Mann–Whitney *U* test).

In order to analyze the difference of immune cell proportion between AC003092.1_H and AC003092.1_L, we applied CIBERSORTx, which is a deconvolution algorithm based on gene expression (Newman et al., 2019). CIBERSORTx was combined with LM22, which have 547 gene expression characteristics to distinguish 22 immune cell subsets and define *p* value of the proportions of immune cell subsets. One of the GBM patients in AC003092.1_L was excluded because of *p* > 0.05. According to the result of CIBERSORTx, the proportion of immune cells between AC003092.1_H and AC003092.1_L varies widely (Figure 4A). Monocytes and neutrophils had a higher proportion in the AC003092.1_H, whereas the percentages of naive B cell, T cells, follicular helper cells, and macrophages M1 were higher in AC003092.1_L (Mann–Whitney *U* test, *p* < 0.05) (Figure 4B).

### Validation of AC003092.1 and TFPI2 Levels by RT-qPCR

**FIGURE 5:**
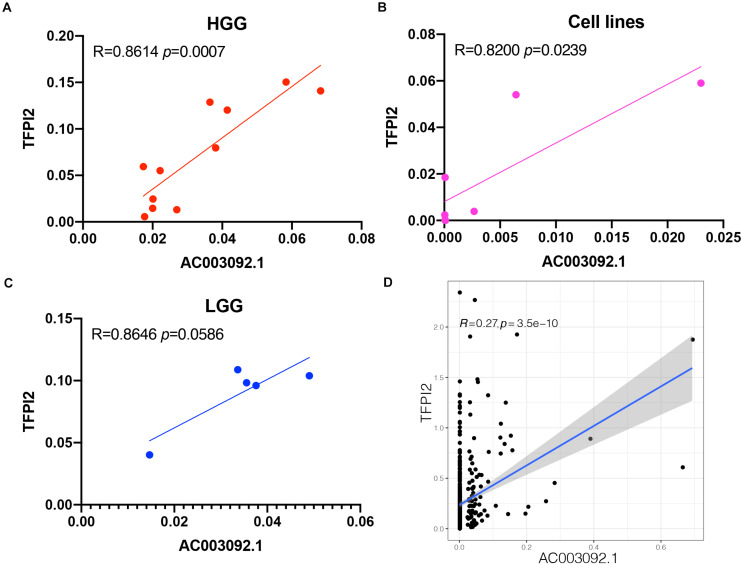
Validation of *AC003092.1* and *TFPI2* levels by RT-qPCR. Scatterplot showing correlation between *AC003092.1* and *TFPI2* levels in HGG **(A)**, GBM cell lines **(B)**, LGG **(C)**, and patients with LGG in TCGA **(D)**.

The correlation between *AC003092.1* and *TFPI2* in 11 patients with high-grade gliomas, 5 patients with low-grade gliomas, and 7 glioblastoma cell lines was investigated using RT-qPCR. In high-grade gliomas and glioblastoma cell lines, there was a significantly positive correlation between *AC003092.1* and *TFPI2* (high-grade gliomas: Spearman rank correlation coefficient *r* = 0.8614, *p* < 0.001; glioblastoma cell lines: Spearman rank correlation coefficient *r* = 0.8200, *p* < 0.05) (Figures 5A,B and Supplementary Figure 1). There was no significant correlation between the two RNAs in low-grade gliomas, the same as the result in 529 low-grade gliomas of TCGA (Spearman rank correlation coefficient *r* = 0.8646, *p* > 0.05) (Figures 5C,D).

## Discussion

eRNA as an emerging class of lncRNAs that could regulate target gene expression to influence biological process has received especially high levels of attention. In our study, we first screened prognosis-related eRNAs from glioblastoma transcriptome data through bioinformatics. Then, we identified *AC003092.1* as an immune-related eRNA by GO and KEGG enrichment analysis. Moreover, we quantified the immune signature by ssGSEA and ESTIMATE. And we depicted the landscape of immune cell fractions in different groups. Finally, we validated the *AC003092.1* and *TFPI2* transcript levels in high-grade gliomas, low-grade gliomas, and GBM cell lines.

Instead of proliferation and differentiation, lncRNA had the ability to activate immune cells, including T cells, B cells, macrophages, monocytes, and so on (Satpathy and Chang, 2015). Lnc-Lsm3b could bind to retinoic acid–inducible gene-I to terminate type I interferon production to restrict innate immune response (Jiang et al., 2018). Moreover, lncRNA could affect tumor immune. LncRNA NKILA promoted tumor immune evasion by inhibiting nuclear factor κB activity to regulate T-cell sensitivity to activation-induced cell death (Huang et al., 2018). Recently, many studies focus on the function of lncRNAs in glioma. And some immune-related lncRNAs had been identified as genes that could regulate glioma microenvironment (Wang et al., 2018; Zhou et al., 2018; Li and Meng, 2019).

*AC003092.1* as a functional lncRNA was reported by Xu et al. (2018). They found AC*003092.1* located at about 32 kb from *TFPI2* and revealed that *TFPI2* was the target gene of *AC003092.1* in temozolomide-resistant glioma cells. In subsequent study, Xu et al. demonstrated that *AC003092.1* could inhibit the function of *miR-195* to upregulate expression of *TFPI2* and promote TMZ-induced apoptosis. In the present study, instead of chemosensitivity, *AC003092.1* was potentially associated with the formation of glioblastoma immunosuppressive microenvironment. In function enrichment analysis, we found that *AC003092.1*-related genes were mainly enriched in immune-associated terms and pathways. And the immune-related feature showed that immune cell types, functions, and pathways were enriched in AC003092.1_H. Even so, CD8^+^ T cells in AC003092.1_H were exhausted because the *PD-L1* and *TIM-3* were expressed highly. And in previous study of transcriptional landscape of eRNA, the authors found that six checkpoints were correlated with eRNAs in at least five cancer types (Zhang et al., 2019). It had been demonstrated that colorectal cancer–associated transcript 1-long isoform (*CCAT1-L*) as a super-enhancer RNA (a cluster of eRNA) could bind at *MYC* to induce many pro-proliferative genes and maintain cancer cell survival by exempting from immune surveillance and the antitumor immune response (Cotterman et al., 2008; Kim et al., 2017). Thus, eRNA had putative interaction with immune checkpoints and could be a potential therapeutic target in malignancy treatment.

GO:0002274: myeloid leukocyte activation was the most significant terms that *AC003092.1*-related genes enriched. At the same time, we found that the proportions of monocytes, macrophages, and neutrophils were notably different between AC003092.1_H and AC003092.1_L.

Tissue factor pathway inhibitor 2, a 32-kDa matrix-associated Kunitz-type serine protease inhibitor, was encoded by the *TFPI2* gene. The expression of TFPI2 was negatively correlated with tumor invasion and proliferation (Sierko et al., 2007). Thus, *TFPI2* was regarded as a tumor suppressor gene. *TFPI2* was also related to the occurrence and development of glioma. In glioblastoma, *AGAP2-AS1* could epigenetically silence *TFPI2* expression by binding to *EZH2* and *LSD1* to promote GBM (Luo et al., 2019). TFPI2 had conserved C-terminal region. Kasetty et al. (2016) demonstrated that the C-terminal TFPI-2–derived peptides from different vertebrates could exert antibacterial effects through the complement activation. The neutrophil elastase could generate a C-terminal *TFPI-2* fragment, which interacted with bacteria to inhibit microorganism growth (Papareddy et al., 2012). The expression of TFPI2 in human umbilical vein endothelial cell was significantly upregulated after stimulation with inflammatory mediators such as Phorbol-12-myristate-13-acetate (PMA), lipopolysaccharide, and tumor necrosis factor α (Iino et al., 1998). Research had shown that recombinant TFPI-2 could promote U937-derived macrophage apoptosis by upregulation of Fas/FsaL (Pan et al., 2011). Papareddy et al. (2012) demonstrated that *TFPI2* existed in skin and would be upregulated during the wounding. These researches revealed that TFPI2 could participate in the immune response in different ways. So, we hypothesized that although *AC003092.1* was a non-coding RNA, it could upregulate *TFPI2* expression to affect GBM immune response.

## Conclusion

According to our study, we found that *AC003092.1* was an immune-related gene in glioblastoma patients, and the genes that correlated to *AC003092.1* are enriched in immune-associated terms and pathways especially in myeloid leukocyte activation. Immune cell types, functions, and pathways were enriched in AC003092.1_H by ssGSEA. And the present study indicated that *AC003092.1* had a close relationship with monocytes, macrophages, and neutrophils. The results of qRT-PCR validated the *AC003092.1* and *TFPI2* levels in high-grade glioma and glioblastoma cell lines. This provides a new idea for studying the immunosuppressive microenvironment of glioblastoma.

## Data Availability Statement

Publicly available datasets were analyzed in this study. This data can be found here: https://portal.gdc.cancer.gov/.

## Ethics Statement

The studies involving human participants were reviewed and approved by the Ethics Committee of Sun Yat-sen University Cancer Center. The patients/participants provided their written informed consent to participate in this study.

## Author Contributions

X-YG, SZ, and Z-NW participated in the design of the study, performed the statistical analysis, interpretation of data, and drafted the manuscript. HD, YW, and J-JD collected and organized the data. TX, J-YZ, and G-HZ participated in the design of the study and performed the statistical analysis. LL, RC, H-RH, and JL carried out the qRT-PCR. Z-QH and Y-GM conceived of the study, and revised and approved the final manuscript. All authors read and approved the final manuscript.

## Conflict of Interest

The authors declare that the research was conducted in the absence of any commercial or financial relationships that could be construed as a potential conflict of interest.

## Footnotes

1 https://metascape.org/

2 https://cibersortx.stanford.edu/

## Supplementary Material

Supplementary Figure 1 The level of expression of AC003092.1 and TFPI2 in each single cell line.
