# Supplementary material for: Immunogenomic Profiling Demonstrate AC003092.1 as an Immune-Related eRNA in Glioblastoma Multiforme
Source: Front Genet. 2021 Mar 18;12:633812. doi: 10.3389/fgene.2021.633812 (PMC8012670; doi:10.3389/fgene.2021.633812)
Supplement: Supplementary file 1 [file Table_1.DOCX]

| Symbol | Overall Survival Analysis  Log-Rank p-Value | Symbol | Overall Survival Analysis  Log-Rank p-Value | Symbol | Overall Survival Analysis,  Log-Rank p-Value |
| --- | --- | --- | --- | --- | --- |
| AL356215.1 | 0.02270796 | PROX1-AS1 | 0.01963744 | ALDH3B1 | 0.00828763 |
| LINC01285 | 0.02215085 | CHST12 | 0.02509646 | AC078785.1 | 0.01551716 |
| LINC01615 | 0.02529253 | AC073316.2 | 0.0480653 | LINC01529 | 0.02333289 |
| LINC02495 | 0.02298029 | AF015262.1 | 0.02863629 | AP000424.1 | 0.01005455 |
| LINC00886 | 0.00379627 | LINC02324 | 0.01752688 | LRRC8C-DT | 0.02855255 |
| LEF1-AS1 | 0.00043435 | CYP1B1-AS1 | 0.01464708 | AP002761.1 | 0.03447617 |
| AC015909.1 | 0.01863931 | LINC02773 | 0.00608834 | EDNRB-AS1 | 0.01663754 |
| LINC02036 | 0.00400721 | APELA | 0.03084809 | LY6E-DT | 0.01827051 |
| LINC01111 | 0.04595298 | SPRY4-AS1 | 0.00484478 | AC107223.1 | 0.00312018 |
| SLC44A3-AS1 | 0.02913091 | AC125613.1 | 0.04665323 | AL162411.1 | 0.03847916 |
| ZNF337-AS1 | 0.00670872 | LINC02026 | 0.02113594 | AL158151.1 | 0.00590551 |
| AC069281.1 | 0.03137853 | AL390198.1 | 0.02557166 | AC018866.2 | 0.00082702 |
| AL365259.1 | 0.04065605 | LINC01426 | 0.02473149 | ZMIZ1-AS1 | 1.16E-05 |
| AL355304.1 | 0.03326481 | AC022182.1 | 0.03699002 | AC003092.1 | 0.00577164 |
| LINC01349 | 0.02326706 | IGHA2 | 0.02224705 | LINC00665 | 0.03600934 |
| GCC2-AS1 | 0.00188822 | MIR217HG | 0.01896355 | AL355607.1 | 0.02026224 |
| AP003469.2 | 0.00731351 | AL021707.2 | 0.0312981 | LINC02154 | 0.04628873 |
| HOTAIR | 0.04569144 | AL021937.1 | 0.00397125 | MATN1-AS1 | 0.00148096 |
| CRNDE | 0.00643793 | RPS10P7 | 0.01853641 | AP001189.3 | 0.03737019 |
| AC092164.1 | 0.01054089 | AC083864.2 | 0.02748067 | HOXC-AS3 | 0.00788985 |
| OSMR-AS1 | 0.01893611 | LINC01088 | 0.01687059 | LINC02062 | 0.04318186 |
| LINC01574 | 0.00501611 | FZD4-DT | 0.03702395 | AP003472.2 | 0.04623358 |
| MYOSLID | 0.00880225 | AP003555.1 | 0.03078943 | AC013287.1 | 0.00804998 |
| AC004923.4 | 0.01014952 |  |  |  |  |

Table S1. List of overall survival associated lncRNAs derived from enhancers.
