# Supplementary material for: Immunogenomic Profiling Demonstrate AC003092.1 as an Immune-Related eRNA in Glioblastoma Multiforme
Source: Front Genet. 2021 Mar 18;12:633812. doi: 10.3389/fgene.2021.633812 (PMC8012670; doi:10.3389/fgene.2021.633812)
Supplement: Supplementary file 2 [file Table_2.DOCX]

Table S2. List of genes significantly correlated to AC003092.1.

| eRNA | gene | cor | pvalue |
| --- | --- | --- | --- |
| AC003092.1 | ADTRP | 0.439 | 5.46E-09 |
| AC003092.1 | UPP1 | 0.544 | 8.91E-14 |
| AC003092.1 | SPINK1 | 0.535 | 2.79E-13 |
| AC003092.1 | FCGR2B | 0.535 | 2.60E-13 |
| AC003092.1 | LDHA | 0.417 | 3.68E-08 |
| AC003092.1 | PLP2 | 0.414 | 4.70E-08 |
| AC003092.1 | PLK3 | 0.458 | 9.63E-10 |
| AC003092.1 | ABCA1 | 0.402 | 1.30E-07 |
| AC003092.1 | ANKRD1 | 0.42 | 3.01E-08 |
| AC003092.1 | PTGS2 | 0.555 | 2.04E-14 |
| AC003092.1 | MMP8 | 0.408 | 7.95E-08 |
| AC003092.1 | SLPI | 0.48 | 1.18E-10 |
| AC003092.1 | CLEC5A | 0.468 | 4.02E-10 |
| AC003092.1 | AC022509.1 | 0.445 | 3.28E-09 |
| AC003092.1 | ANTXR2 | 0.404 | 1.04E-07 |
| AC003092.1 | RARRES1 | 0.422 | 2.52E-08 |
| AC003092.1 | CFB | 0.423 | 2.32E-08 |
| AC003092.1 | AC099548.2 | 0.407 | 8.25E-08 |
| AC003092.1 | CASP4 | 0.416 | 4.04E-08 |
| AC003092.1 | SLC66A3 | 0.474 | 2.17E-10 |
| AC003092.1 | RAC2 | 0.435 | 8.12E-09 |
| AC003092.1 | TNFAIP3 | 0.474 | 2.07E-10 |
| AC003092.1 | ACAP1 | 0.406 | 8.81E-08 |
| AC003092.1 | AL034397.3 | 0.446 | 3.13E-09 |
| AC003092.1 | ADAM8 | 0.507 | 6.57E-12 |
| AC003092.1 | TREM1 | 0.62 | 1.75E-18 |
| AC003092.1 | AL355607.2 | 0.535 | 2.52E-13 |
| AC003092.1 | CTSC | 0.42 | 2.98E-08 |
| AC003092.1 | VDR | 0.518 | 1.91E-12 |
| AC003092.1 | PTX3 | 0.566 | 5.33E-15 |
| AC003092.1 | IQGAP1 | 0.409 | 7.32E-08 |
| AC003092.1 | BX640514.2 | 0.486 | 6.46E-11 |
| AC003092.1 | CTBS | 0.419 | 3.25E-08 |
| AC003092.1 | ANXA2P3 | 0.411 | 6.22E-08 |
| AC003092.1 | HSPA5 | 0.405 | 9.81E-08 |
| AC003092.1 | AC078850.1 | 0.467 | 4.06E-10 |
| AC003092.1 | AKR1B10 | 0.435 | 8.00E-09 |
| AC003092.1 | PI3 | 0.661 | 1.31E-21 |
| AC003092.1 | CATSPER1 | 0.477 | 1.55E-10 |
| AC003092.1 | LILRA5 | 0.453 | 1.59E-09 |
| AC003092.1 | HRH1 | 0.417 | 3.86E-08 |
| AC003092.1 | LAIR1 | 0.42 | 2.85E-08 |
| AC003092.1 | STC1 | 0.484 | 7.90E-11 |
| AC003092.1 | CD300E | 0.569 | 3.52E-15 |
| AC003092.1 | LUCAT1 | 0.603 | 2.43E-17 |
| AC003092.1 | RHOH | 0.474 | 2.23E-10 |
| AC003092.1 | ENTHD1 | 0.533 | 3.34E-13 |
| AC003092.1 | CPD | 0.501 | 1.29E-11 |
| AC003092.1 | AC112721.2 | 0.45 | 2.16E-09 |
| AC003092.1 | LINC02274 | 0.454 | 1.48E-09 |
| AC003092.1 | MCTP2 | 0.446 | 3.13E-09 |
| AC003092.1 | NOD2 | 0.46 | 8.70E-10 |
| AC003092.1 | TPP1 | 0.415 | 4.58E-08 |
| AC003092.1 | RETN | 0.543 | 1.04E-13 |
| AC003092.1 | UAP1 | 0.478 | 1.46E-10 |
| AC003092.1 | ANXA2P2 | 0.481 | 1.07E-10 |
| AC003092.1 | ANGPTL4 | 0.487 | 5.44E-11 |
| AC003092.1 | AC245128.3 | 0.416 | 4.20E-08 |
| AC003092.1 | IL1RN | 0.467 | 4.39E-10 |
| AC003092.1 | CD300LB | 0.407 | 8.29E-08 |
| AC003092.1 | TLR2 | 0.445 | 3.36E-09 |
| AC003092.1 | HAMP | 0.427 | 1.65E-08 |
| AC003092.1 | CXCL2 | 0.434 | 9.03E-09 |
| AC003092.1 | NAMPT | 0.561 | 9.71E-15 |
| AC003092.1 | SAA1 | 0.423 | 2.21E-08 |
| AC003092.1 | PTPN2 | 0.441 | 4.93E-09 |
| AC003092.1 | TMEM70 | 0.41 | 6.81E-08 |
| AC003092.1 | SAA2 | 0.481 | 1.06E-10 |
| AC003092.1 | UGCG | 0.454 | 1.40E-09 |
| AC003092.1 | HIF1A-AS3 | 0.437 | 6.57E-09 |
| AC003092.1 | STAB1 | 0.461 | 7.82E-10 |
| AC003092.1 | AC087645.2 | 0.431 | 1.11E-08 |
| AC003092.1 | LRG1 | 0.418 | 3.31E-08 |
| AC003092.1 | CCL7 | 0.66 | 1.67E-21 |
| AC003092.1 | WDR45P1 | 0.407 | 8.43E-08 |
| AC003092.1 | RNASE2 | 0.551 | 3.47E-14 |
| AC003092.1 | AC100849.1 | 0.404 | 1.09E-07 |
| AC003092.1 | TLCD2 | 0.423 | 2.18E-08 |
| AC003092.1 | TNFAIP8 | 0.483 | 8.90E-11 |
| AC003092.1 | KRT80 | 0.463 | 5.99E-10 |
| AC003092.1 | MAP3K8 | 0.502 | 1.18E-11 |
| AC003092.1 | FCGR3A | 0.439 | 5.81E-09 |
| AC003092.1 | ZNF267 | 0.462 | 6.96E-10 |
| AC003092.1 | ITGAM | 0.416 | 4.08E-08 |
| AC003092.1 | MMP7 | 0.49 | 4.40E-11 |
| AC003092.1 | LACTB | 0.436 | 7.57E-09 |
| AC003092.1 | SLC2A3 | 0.534 | 2.89E-13 |
| AC003092.1 | CFI | 0.52 | 1.53E-12 |
| AC003092.1 | S100A4 | 0.41 | 6.83E-08 |
| AC003092.1 | DYNLT3 | 0.441 | 4.93E-09 |
| AC003092.1 | ADPGK | 0.408 | 8.08E-08 |
| AC003092.1 | IL4R | 0.446 | 3.13E-09 |
| AC003092.1 | SIGLEC7 | 0.418 | 3.46E-08 |
| AC003092.1 | PCED1B-AS1 | 0.442 | 4.51E-09 |
| AC003092.1 | MGST1 | 0.412 | 5.57E-08 |
| AC003092.1 | RUNX2 | 0.417 | 3.66E-08 |
| AC003092.1 | COL6A3 | 0.481 | 1.06E-10 |
| AC003092.1 | CHI3L1 | 0.481 | 1.01E-10 |
| AC003092.1 | PTPN22 | 0.406 | 9.09E-08 |
| AC003092.1 | TMBIM1 | 0.408 | 8.02E-08 |
| AC003092.1 | FCGR2A | 0.516 | 2.56E-12 |
| AC003092.1 | TNFRSF1B | 0.447 | 2.68E-09 |
| AC003092.1 | RNASE3 | 0.453 | 1.57E-09 |
| AC003092.1 | KYNU | 0.515 | 2.82E-12 |
| AC003092.1 | P4HA2 | 0.441 | 4.74E-09 |
| AC003092.1 | SPAG4 | 0.482 | 9.16E-11 |
| AC003092.1 | CHI3L2 | 0.451 | 1.92E-09 |
| AC003092.1 | SLC16A10 | 0.428 | 1.53E-08 |
| AC003092.1 | AC145676.1 | 0.437 | 6.86E-09 |
| AC003092.1 | AQP9 | 0.575 | 1.45E-15 |
| AC003092.1 | NFKBIZ | 0.545 | 7.65E-14 |
| AC003092.1 | CD163 | 0.573 | 1.91E-15 |
| AC003092.1 | CSTA | 0.475 | 1.94E-10 |
| AC003092.1 | BCL3 | 0.537 | 2.14E-13 |
| AC003092.1 | CPA4 | 0.435 | 8.07E-09 |
| AC003092.1 | LILRB2 | 0.421 | 2.62E-08 |
| AC003092.1 | CXCL8 | 0.651 | 9.31E-21 |
| AC003092.1 | MAN1A1 | 0.48 | 1.22E-10 |
| AC003092.1 | CD200R1 | 0.404 | 1.03E-07 |
| AC003092.1 | CA12 | 0.431 | 1.15E-08 |
| AC003092.1 | ELK3 | 0.411 | 5.99E-08 |
| AC003092.1 | GLRX | 0.464 | 5.86E-10 |
| AC003092.1 | IKBIP | 0.429 | 1.31E-08 |
| AC003092.1 | PLAUR | 0.589 | 2.01E-16 |
| AC003092.1 | CEACAM4 | 0.424 | 2.08E-08 |
| AC003092.1 | KMO | 0.404 | 1.04E-07 |
| AC003092.1 | AC022092.1 | 0.446 | 3.12E-09 |
| AC003092.1 | BATF | 0.425 | 1.87E-08 |
| AC003092.1 | AL355922.1 | 0.487 | 5.82E-11 |
| AC003092.1 | C15orf48 | 0.514 | 3.07E-12 |
| AC003092.1 | SYTL2 | 0.46 | 8.32E-10 |
| AC003092.1 | SAT1 | 0.419 | 3.18E-08 |
| AC003092.1 | CRYBG1 | 0.413 | 5.05E-08 |
| AC003092.1 | PTGES | 0.484 | 7.49E-11 |
| AC003092.1 | GPR84 | 0.523 | 1.10E-12 |
| AC003092.1 | AC090796.1 | 0.547 | 6.25E-14 |
| AC003092.1 | GPX8 | 0.416 | 3.95E-08 |
| AC003092.1 | SRPX2 | 0.406 | 9.43E-08 |
| AC003092.1 | F13A1 | 0.57 | 3.06E-15 |
| AC003092.1 | FCGBP | 0.469 | 3.59E-10 |
| AC003092.1 | MYOSLID | 0.439 | 5.94E-09 |
| AC003092.1 | PF4V1 | 0.458 | 9.98E-10 |
| AC003092.1 | SPP1 | 0.455 | 1.28E-09 |
| AC003092.1 | RDH10 | 0.525 | 8.34E-13 |
| AC003092.1 | ST8SIA4 | 0.497 | 2.05E-11 |
| AC003092.1 | KRT16 | 0.432 | 1.07E-08 |
| AC003092.1 | NAMPTP3 | 0.442 | 4.18E-09 |
| AC003092.1 | LRRC15 | 0.499 | 1.60E-11 |
| AC003092.1 | AC061992.2 | 0.56 | 1.12E-14 |
| AC003092.1 | LIMS1 | 0.423 | 2.18E-08 |
| AC003092.1 | INSIG2 | 0.433 | 9.89E-09 |
| AC003092.1 | MS4A4A | 0.486 | 6.23E-11 |
| AC003092.1 | SERPINA1 | 0.473 | 2.48E-10 |
| AC003092.1 | MFSD1 | 0.419 | 3.10E-08 |
| AC003092.1 | LRRFIP1 | 0.405 | 9.97E-08 |
| AC003092.1 | GCNT1 | 0.441 | 4.70E-09 |
| AC003092.1 | AC078883.1 | 0.443 | 4.01E-09 |
| AC003092.1 | ANXA2 | 0.515 | 2.62E-12 |
| AC003092.1 | MPZL3 | 0.412 | 5.87E-08 |
| AC003092.1 | HILPDA | 0.454 | 1.52E-09 |
| AC003092.1 | TGM2 | 0.437 | 6.55E-09 |
| AC003092.1 | YBX3 | 0.42 | 2.89E-08 |
| AC003092.1 | SPOCD1 | 0.483 | 8.35E-11 |
| AC003092.1 | IL1B | 0.452 | 1.71E-09 |
| AC003092.1 | DPYD | 0.499 | 1.63E-11 |
| AC003092.1 | CSF3 | 0.604 | 2.20E-17 |
| AC003092.1 | AC099509.1 | 0.518 | 1.87E-12 |
| AC003092.1 | SMIM25 | 0.524 | 9.36E-13 |
| AC003092.1 | FAS | 0.427 | 1.65E-08 |
| AC003092.1 | BIRC3 | 0.593 | 1.11E-16 |
| AC003092.1 | HMGA2 | 0.445 | 3.39E-09 |
| AC003092.1 | OSMR | 0.467 | 4.07E-10 |
| AC003092.1 | SERPINB2 | 0.423 | 2.28E-08 |
| AC003092.1 | AC092484.1 | 0.46 | 8.29E-10 |
| AC003092.1 | BDKRB2 | 0.456 | 1.26E-09 |
| AC003092.1 | LINC01705 | 0.499 | 1.58E-11 |
| AC003092.1 | TNFSF14 | 0.501 | 1.35E-11 |
| AC003092.1 | IL31RA | 0.429 | 1.35E-08 |
| AC003092.1 | IL13RA1 | 0.466 | 4.72E-10 |
| AC003092.1 | ATL3 | 0.414 | 4.96E-08 |
| AC003092.1 | DYRK3 | 0.414 | 4.82E-08 |
| AC003092.1 | PPP1R18 | 0.427 | 1.63E-08 |
| AC003092.1 | SIGLEC9 | 0.501 | 1.26E-11 |
| AC003092.1 | MYOF | 0.424 | 2.02E-08 |
| AC003092.1 | RCAN1 | 0.426 | 1.80E-08 |
| AC003092.1 | MCEMP1 | 0.56 | 1.07E-14 |
| AC003092.1 | CLDN23 | 0.419 | 3.08E-08 |
| AC003092.1 | BHLHE40 | 0.461 | 7.21E-10 |
| AC003092.1 | SRGN | 0.437 | 7.06E-09 |
| AC003092.1 | ADM | 0.405 | 1.01E-07 |
| AC003092.1 | RAB13 | 0.407 | 8.65E-08 |
| AC003092.1 | PYGL | 0.424 | 2.01E-08 |
| AC003092.1 | IL11 | 0.561 | 9.49E-15 |
| AC003092.1 | VNN3 | 0.492 | 3.51E-11 |
| AC003092.1 | CCL20 | 0.654 | 5.25E-21 |
| AC003092.1 | SOCS3 | 0.607 | 1.37E-17 |
| AC003092.1 | C1orf162 | 0.46 | 8.26E-10 |
| AC003092.1 | S100A10 | 0.468 | 3.89E-10 |
| AC003092.1 | TMED5 | 0.443 | 4.08E-09 |
| AC003092.1 | GCLM | 0.52 | 1.60E-12 |
| AC003092.1 | REXO2 | 0.472 | 2.55E-10 |
| AC003092.1 | AMPD3 | 0.467 | 4.09E-10 |
| AC003092.1 | PRSS23 | 0.422 | 2.42E-08 |
| AC003092.1 | ABCC3 | 0.408 | 7.64E-08 |
| AC003092.1 | EREG | 0.55 | 4.01E-14 |
| AC003092.1 | SOD2 | 0.499 | 1.58E-11 |
| AC003092.1 | ADAMTS14 | 0.407 | 8.36E-08 |
| AC003092.1 | NAMPTP1 | 0.526 | 8.19E-13 |
| AC003092.1 | LINC01127 | 0.441 | 4.67E-09 |
| AC003092.1 | MMP3 | 0.406 | 8.96E-08 |
| AC003092.1 | AC004847.1 | 0.449 | 2.33E-09 |
| AC003092.1 | CACNA2D4 | 0.504 | 9.00E-12 |
| AC003092.1 | IBSP | 0.472 | 2.69E-10 |
| AC003092.1 | CTSL | 0.577 | 1.06E-15 |
| AC003092.1 | ALG2 | 0.416 | 4.19E-08 |
| AC003092.1 | SOAT1 | 0.43 | 1.27E-08 |
| AC003092.1 | NABP1 | 0.466 | 4.72E-10 |
| AC003092.1 | AC026310.2 | 0.557 | 1.63E-14 |
| AC003092.1 | CD14 | 0.49 | 4.14E-11 |
| AC003092.1 | ATP6V0D2 | 0.404 | 1.04E-07 |
| AC003092.1 | SERPINE1 | 0.531 | 4.14E-13 |
| AC003092.1 | ACPP | 0.475 | 2.00E-10 |
| AC003092.1 | PTPN7 | 0.42 | 2.98E-08 |
| AC003092.1 | GPR160 | 0.422 | 2.52E-08 |
| AC003092.1 | NPC2 | 0.496 | 2.14E-11 |
| AC003092.1 | C1R | 0.489 | 4.46E-11 |
| AC003092.1 | FCAR | 0.426 | 1.81E-08 |
| AC003092.1 | LINC02392 | 0.486 | 6.17E-11 |
| AC003092.1 | LGALS12 | 0.472 | 2.71E-10 |
| AC003092.1 | THBS1 | 0.435 | 7.76E-09 |
| AC003092.1 | PLA2G2A | 0.413 | 5.17E-08 |
| AC003092.1 | SDC4 | 0.445 | 3.32E-09 |
| AC003092.1 | CYP1B1 | 0.47 | 3.18E-10 |
| AC003092.1 | MET | 0.515 | 2.72E-12 |
| AC003092.1 | CCR1 | 0.403 | 1.20E-07 |
| AC003092.1 | SELE | 0.403 | 1.18E-07 |
| AC003092.1 | CTSZ | 0.43 | 1.24E-08 |
| AC003092.1 | GNS | 0.431 | 1.11E-08 |
| AC003092.1 | SUMF1 | 0.441 | 4.58E-09 |
| AC003092.1 | VENTX | 0.467 | 4.31E-10 |
| AC003092.1 | IL1R1 | 0.533 | 3.36E-13 |
| AC003092.1 | C1RL | 0.537 | 2.11E-13 |
| AC003092.1 | CLEC2B | 0.501 | 1.27E-11 |
| AC003092.1 | MMP19 | 0.589 | 1.96E-16 |
| AC003092.1 | FOLR3 | 0.457 | 1.08E-09 |
| AC003092.1 | ALOX5AP | 0.507 | 6.97E-12 |
| AC003092.1 | S100A8 | 0.559 | 1.36E-14 |
| AC003092.1 | CCR2 | 0.424 | 2.07E-08 |
| AC003092.1 | CXCL1 | 0.533 | 3.57E-13 |
| AC003092.1 | BET1 | 0.411 | 6.24E-08 |
| AC003092.1 | EVA1A | 0.404 | 1.04E-07 |
| AC003092.1 | CD70 | 0.433 | 9.85E-09 |
| AC003092.1 | TFPI2 | 0.596 | 7.16E-17 |
| AC003092.1 | MRC1 | 0.469 | 3.52E-10 |
| AC003092.1 | MAPK13 | 0.484 | 8.20E-11 |
| AC003092.1 | IL36B | 0.602 | 2.97E-17 |
| AC003092.1 | ERO1A | 0.517 | 2.08E-12 |
| AC003092.1 | IL21-AS1 | 0.415 | 4.23E-08 |
| AC003092.1 | SERPINB1 | 0.425 | 1.84E-08 |
| AC003092.1 | B4GALT1 | 0.436 | 7.23E-09 |
| AC003092.1 | DSE | 0.488 | 5.41E-11 |
| AC003092.1 | GPRC5A | 0.469 | 3.52E-10 |
| AC003092.1 | IL18R1 | 0.496 | 2.20E-11 |
| AC003092.1 | TSLP | 0.436 | 7.46E-09 |
| AC003092.1 | RNF144B | 0.416 | 4.11E-08 |
| AC003092.1 | CCL2 | 0.565 | 5.70E-15 |
| AC003092.1 | LYVE1 | 0.499 | 1.55E-11 |
| AC003092.1 | ALDH3B1 | 0.412 | 5.45E-08 |
| AC003092.1 | MYO1G | 0.533 | 3.42E-13 |
| AC003092.1 | LTBP2 | 0.466 | 4.85E-10 |
| AC003092.1 | MMP10 | 0.47 | 3.15E-10 |
| AC003092.1 | CXCL3 | 0.637 | 1.02E-19 |
| AC003092.1 | RNF149 | 0.411 | 6.12E-08 |
| AC003092.1 | CD300A | 0.445 | 3.37E-09 |
| AC003092.1 | P4HA1 | 0.454 | 1.41E-09 |
| AC003092.1 | CCL13 | 0.519 | 1.75E-12 |
| AC003092.1 | CYTIP | 0.409 | 7.14E-08 |
| AC003092.1 | S100A11 | 0.451 | 1.93E-09 |
| AC003092.1 | SLC6A6 | 0.421 | 2.68E-08 |
| AC003092.1 | VSIG4 | 0.47 | 3.09E-10 |
| AC003092.1 | AL356414.1 | 0.411 | 6.27E-08 |
| AC003092.1 | ELL2 | 0.489 | 4.79E-11 |
| AC003092.1 | RPSAP52 | 0.473 | 2.35E-10 |
| AC003092.1 | IL1A | 0.454 | 1.50E-09 |
| AC003092.1 | MAN1C1 | 0.401 | 1.35E-07 |
| AC003092.1 | ORM1 | 0.542 | 1.12E-13 |
| AC003092.1 | IL7R | 0.521 | 1.40E-12 |
| AC003092.1 | MVP | 0.405 | 9.58E-08 |
| AC003092.1 | DSC2 | 0.426 | 1.76E-08 |
| AC003092.1 | CAPZA1 | 0.424 | 2.10E-08 |
| AC003092.1 | LRRC25 | 0.423 | 2.32E-08 |
| AC003092.1 | AC123595.1 | 0.42 | 3.03E-08 |
| AC003092.1 | CXCL6 | 0.612 | 6.58E-18 |
| AC003092.1 | THBD | 0.539 | 1.70E-13 |
| AC003092.1 | FPR1 | 0.429 | 1.32E-08 |
| AC003092.1 | SIGLEC10 | 0.421 | 2.76E-08 |
| AC003092.1 | AC004988.1 | 0.428 | 1.45E-08 |
| AC003092.1 | RRAS | 0.449 | 2.26E-09 |
| AC003092.1 | CXCL5 | 0.58 | 7.30E-16 |
| AC003092.1 | CLRN3 | 0.411 | 6.31E-08 |
| AC003092.1 | LOX | 0.579 | 8.03E-16 |
| AC003092.1 | PLB1 | 0.443 | 3.91E-09 |
| AC003092.1 | ITGA5 | 0.428 | 1.51E-08 |
| AC003092.1 | ANXA2P1 | 0.423 | 2.29E-08 |
| AC003092.1 | FCER1G | 0.447 | 2.77E-09 |
| AC003092.1 | ZC3H12A | 0.503 | 1.07E-11 |
| AC003092.1 | FOSL2 | 0.471 | 2.91E-10 |
| AC003092.1 | CEBPB | 0.477 | 1.55E-10 |
| AC003092.1 | JAK3 | 0.438 | 6.21E-09 |
| AC003092.1 | HEXB | 0.419 | 3.28E-08 |
| AC003092.1 | S100A9 | 0.557 | 1.61E-14 |
| AC003092.1 | CAV1 | 0.478 | 1.44E-10 |
| AC003092.1 | ALOX5 | 0.454 | 1.42E-09 |
| AC003092.1 | HMOX1 | 0.451 | 2.01E-09 |
| AC003092.1 | LINC02454 | 0.428 | 1.45E-08 |
| AC003092.1 | C1S | 0.518 | 1.94E-12 |
| AC003092.1 | LILRB3 | 0.444 | 3.65E-09 |
| AC003092.1 | PLOD2 | 0.463 | 6.28E-10 |
| AC003092.1 | PDK3 | 0.414 | 4.87E-08 |
| AC003092.1 | BACE2 | 0.428 | 1.54E-08 |
| AC003092.1 | PIM1 | 0.417 | 3.83E-08 |
| AC003092.1 | AC007953.1 | 0.414 | 4.94E-08 |
| AC003092.1 | ICAM1 | 0.536 | 2.43E-13 |
| AC003092.1 | LMAN1 | 0.416 | 4.08E-08 |
| AC003092.1 | SLAMF8 | 0.424 | 2.03E-08 |
| AC003092.1 | PAPPA | 0.446 | 2.97E-09 |
| AC003092.1 | ASGR2 | 0.45 | 2.08E-09 |
| AC003092.1 | TREML3P | 0.482 | 9.92E-11 |
| AC003092.1 | WIPI1 | 0.445 | 3.19E-09 |
| AC003092.1 | BCL2A1 | 0.503 | 1.07E-11 |
| AC003092.1 | NDRG1 | 0.487 | 5.47E-11 |
| AC003092.1 | CLCF1 | 0.538 | 1.89E-13 |
| AC003092.1 | OSTF1 | 0.408 | 7.59E-08 |
| AC003092.1 | PROS1 | 0.411 | 5.92E-08 |
| AC003092.1 | CD164 | 0.401 | 1.40E-07 |
| AC003092.1 | KRT7 | 0.487 | 5.73E-11 |
| AC003092.1 | SLC16A3 | 0.423 | 2.20E-08 |
| AC003092.1 | TGFBI | 0.47 | 3.05E-10 |
| AC003092.1 | PPBP | 0.432 | 1.09E-08 |
| AC003092.1 | AP3S1 | 0.407 | 8.47E-08 |
| AC003092.1 | LINC01503 | 0.491 | 3.67E-11 |
| AC003092.1 | MEDAG | 0.429 | 1.35E-08 |
| AC003092.1 | ADGRE2 | 0.46 | 8.62E-10 |
| AC003092.1 | CSF2 | 0.482 | 9.56E-11 |
| AC003092.1 | AC107959.3 | 0.447 | 2.85E-09 |
| AC003092.1 | RBM47 | 0.476 | 1.79E-10 |
| AC003092.1 | COL1A1 | 0.42 | 3.02E-08 |
| AC003092.1 | ZMIZ1-AS1 | 0.468 | 3.68E-10 |
| AC003092.1 | IL1R2 | 0.41 | 6.85E-08 |
| AC003092.1 | PLIN2 | 0.483 | 8.29E-11 |
| AC003092.1 | AHNAK2 | 0.461 | 7.82E-10 |
| AC003092.1 | MARCO | 0.509 | 5.70E-12 |
| AC003092.1 | PPP1R3B | 0.434 | 8.54E-09 |
| AC003092.1 | LINC02154 | 0.518 | 1.98E-12 |
| AC003092.1 | CDCP1 | 0.467 | 4.09E-10 |
| AC003092.1 | CAST | 0.477 | 1.58E-10 |
| AC003092.1 | CR1 | 0.498 | 1.82E-11 |
| AC003092.1 | IL2RA | 0.509 | 5.40E-12 |
| AC003092.1 | LINC00601 | 0.441 | 4.97E-09 |
| AC003092.1 | ABCA13 | 0.441 | 4.83E-09 |
| AC003092.1 | COL8A1 | 0.405 | 9.85E-08 |
| AC003092.1 | ITGA6-AS1 | 0.499 | 1.54E-11 |
| AC003092.1 | TREML2 | 0.524 | 9.58E-13 |
| AC003092.1 | SNX10 | 0.409 | 6.93E-08 |
| AC003092.1 | IER3 | 0.527 | 7.21E-13 |
| AC003092.1 | FOSL1 | 0.511 | 4.44E-12 |
| AC003092.1 | IL15 | 0.419 | 3.28E-08 |
| AC003092.1 | SH2D2A | 0.453 | 1.65E-09 |
| AC003092.1 | CHCHD7 | 0.501 | 1.30E-11 |
| AC003092.1 | RAB27A | 0.468 | 3.95E-10 |
| AC003092.1 | LCN2 | 0.469 | 3.46E-10 |
| AC003092.1 | SLC39A14 | 0.596 | 7.24E-17 |
| AC003092.1 | AL354919.2 | 0.445 | 3.33E-09 |
| AC003092.1 | LY96 | 0.476 | 1.69E-10 |
| AC003092.1 | GLIPR1 | 0.539 | 1.65E-13 |
| AC003092.1 | C5AR1 | 0.516 | 2.43E-12 |
| AC003092.1 | HSD3B7 | 0.454 | 1.43E-09 |
| AC003092.1 | TACSTD2 | 0.434 | 8.92E-09 |
| AC003092.1 | TPM3 | 0.432 | 1.02E-08 |
| AC003092.1 | GPAT3 | 0.468 | 3.80E-10 |
| AC003092.1 | LIF | 0.517 | 2.20E-12 |
| AC003092.1 | IGFBP6 | 0.446 | 2.93E-09 |
| AC003092.1 | GNA15 | 0.539 | 1.64E-13 |
| AC003092.1 | AC093895.2 | 0.508 | 5.72E-12 |
| AC003092.1 | DRAM1 | 0.444 | 3.55E-09 |
| AC003092.1 | NTAN1 | 0.464 | 5.60E-10 |
| AC003092.1 | GIPC2 | 0.434 | 8.88E-09 |
| AC003092.1 | PTP4A2 | 0.459 | 8.96E-10 |
| AC003092.1 | FAM177B | 0.471 | 2.82E-10 |
| AC003092.1 | LINC01605 | 0.439 | 5.62E-09 |
| AC003092.1 | SGMS2 | 0.485 | 6.91E-11 |
| AC003092.1 | SERPINB8 | 0.493 | 3.09E-11 |
| AC003092.1 | NECTIN4 | 0.41 | 6.72E-08 |
| AC003092.1 | CCL18 | 0.437 | 6.78E-09 |
| AC003092.1 | TNFAIP2 | 0.45 | 2.07E-09 |
| AC003092.1 | SLC11A1 | 0.47 | 3.25E-10 |
| AC003092.1 | FAM20C | 0.429 | 1.33E-08 |
| AC003092.1 | CTSB | 0.514 | 3.20E-12 |
| AC003092.1 | CD1D | 0.425 | 1.88E-08 |
| AC003092.1 | PDPN | 0.419 | 3.20E-08 |
| AC003092.1 | MIR3945HG | 0.42 | 2.95E-08 |
| AC003092.1 | EMB | 0.436 | 7.50E-09 |
| AC003092.1 | MMP1 | 0.546 | 6.67E-14 |
| AC003092.1 | AL391903.1 | 0.467 | 4.44E-10 |
| AC003092.1 | IL6 | 0.496 | 2.16E-11 |
| AC003092.1 | TNFRSF1A | 0.403 | 1.17E-07 |
| AC003092.1 | COL13A1 | 0.43 | 1.31E-08 |
| AC003092.1 | PRELID2 | 0.412 | 5.45E-08 |
| AC003092.1 | HK3 | 0.522 | 1.29E-12 |
| AC003092.1 | CD44 | 0.545 | 8.25E-14 |
| AC003092.1 | HIPK3 | 0.408 | 7.93E-08 |
| AC003092.1 | VASN | 0.434 | 8.57E-09 |
| AC003092.1 | FPR2 | 0.591 | 1.45E-16 |
| AC003092.1 | RPLP0P2 | 0.429 | 1.36E-08 |
| AC003092.1 | MME | 0.418 | 3.37E-08 |
| AC003092.1 | TNFRSF9 | 0.457 | 1.14E-09 |
| AC003092.1 | ITK | 0.424 | 2.00E-08 |
| AC003092.1 | TNFRSF11A | 0.45 | 2.10E-09 |
| AC003092.1 | CYTH4 | 0.405 | 9.78E-08 |
| AC003092.1 | TIMP1 | 0.51 | 5.08E-12 |
