# Supplementary material for: Immunogenomic Profiling Demonstrate AC003092.1 as an Immune-Related eRNA in Glioblastoma Multiforme
Source: Front Genet. 2021 Mar 18;12:633812. doi: 10.3389/fgene.2021.633812 (PMC8012670; doi:10.3389/fgene.2021.633812)
Supplement: Supplementary file 4 [file Table_4.DOCX]

| No. | Gender | Age | Clinicopathologic diagnosis | WHO grade |
| --- | --- | --- | --- | --- |
| 1 | Male | 62 | Glioblastoma | Ⅳ |
| 2 | Male | 70 | Glioblastoma | Ⅳ |
| 3 | Male | 67 | Glioblastoma | Ⅳ |
| 4 | Male | 69 | Glioblastoma | Ⅳ |
| 5 | Female | 48 | Glioblastoma | Ⅳ |
| 6 | Male | 34 | Glioblastoma | Ⅳ |
| 7 | Male | 55 | Glioblastoma | Ⅳ |
| 8 | Male | 47 | Glioblastoma | Ⅳ |
| 9 | Male | 47 | Glioblastoma | Ⅳ |
| 10 | Female | 24 | Anaplastic astrocytoma | Ⅲ |
| 11 | Female | 46 | Anaplastic oligodendroglioma | Ⅲ |
| 12 | Female | 56 | Oligodendroglioma | Ⅱ |
| 13 | Female | 54 | Diffuse astrocytoma | Ⅱ |
| 14 | Male | 42 | Diffuse astrocytoma | Ⅱ |
| 15 | Female | 48 | Diffuse astrocytoma | Ⅱ |
| 16 | Male | 49 | Diffuse astrocytoma | Ⅱ |

Table S4. The clinicopathological features of glioma patients from SYSUCC.
