# Supplementary figures and images for: Immunogenomic Profiling Demonstrate AC003092.1 as an Immune-Related eRNA in Glioblastoma Multiforme
Source: Front Genet. 2021 Mar 18;12:633812. doi: 10.3389/fgene.2021.633812 (PMC8012670; doi:10.3389/fgene.2021.633812)

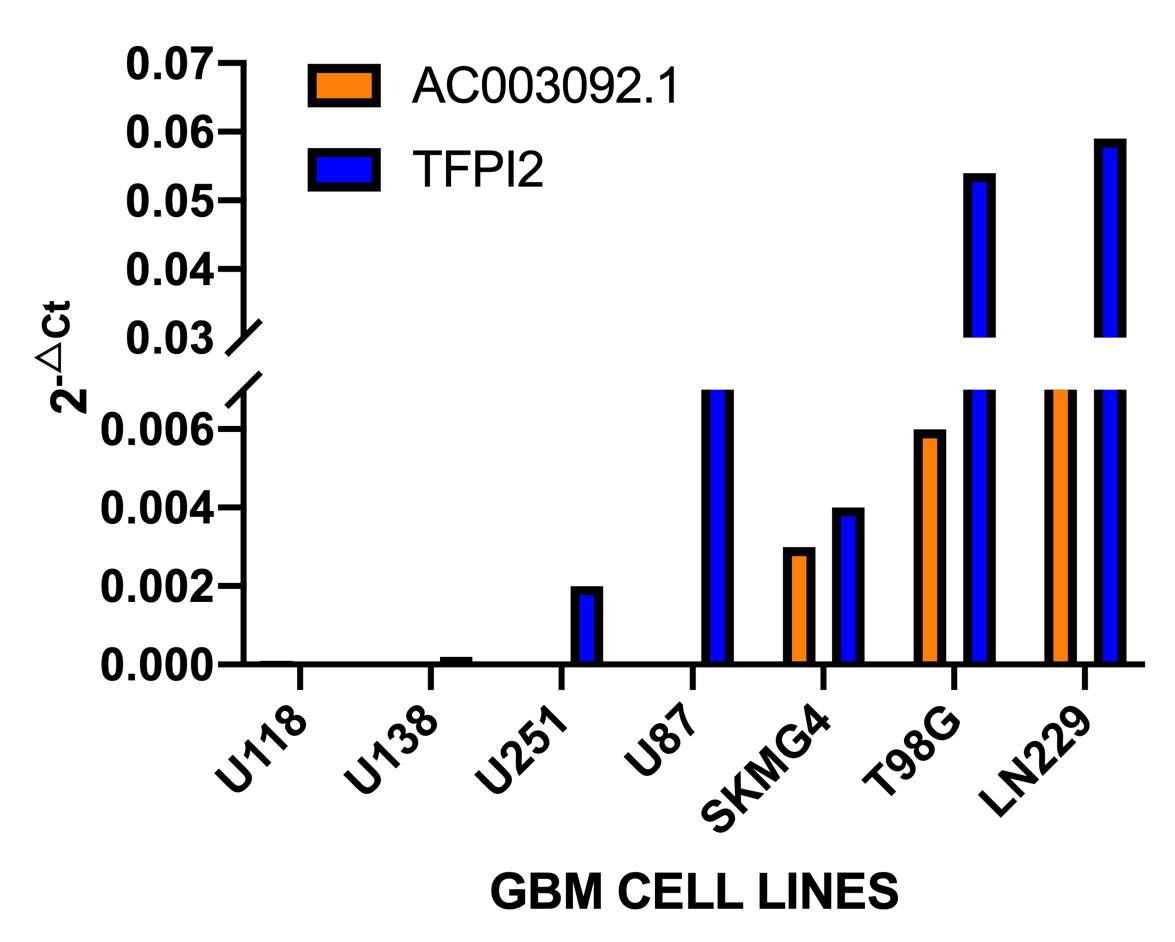

Supplement: Supplementary Figure 1 — The level of expression of AC003092.1 and TFPI2 in each single cell line. [file Image_1.TIFF]
